# Solar Ultraviolet Exposure in Individuals Who Perform Outdoor Sport Activities

**DOI:** 10.1186/s40798-020-00272-9

**Published:** 2020-09-03

**Authors:** Alan Snyder, Manuel Valdebran, David Terrero, Kyle T. Amber, Kristen M. Kelly

**Affiliations:** 1 College of Graduate Studies, Medical University of South Carolina, Charleston, SC USA; 2 Division of Pediatric Dermatology, Department of Dermatology, Medical University of South Carolina, Charleston, SC USA; 3 College of Pharmacy and Pharmaceutical Sciences, University of Toledo, Toledo, OH USA; 4 Department of Dermatology, University of Illinois at Chicago, Chicago, IL USA; 5 Department of Dermatology, University of California, Irvine, Irvine, CA USA

**Keywords:** Sun exposure, Skin cancer, Ultraviolet index, Dosimeter, Outdoor sports, Patient education, Athletes, Mobile technology

## Abstract

**Background:**

Skin cancer is the most common cancer in the USA. Therefore, it is important to review the contribution of ultraviolet radiation (UVR) exposure to skin cancer in individuals with the highest risk. Documenting the relationship between outdoor sports solar ultraviolet exposure and their risk of skin cancer along with appropriate risk mitigation strategies can help inform clinicians of practical information for counseling sun protective behaviors in this population.

**Methods:**

We conducted a review of the current evidence using PubMed to answer the following research questions: (1) How is ultraviolet radiation measured? (2) What is the modern utility of the ultraviolet index in modifying recreational sun protection behaviors? (3) What is the risk of developing skin cancer for outdoor sport participants? (4) What is the prevalence of skin cancer in sport participants? and (5) Is the number of nevi and solar lentigines elevated in outdoor sport participants?

**Results:**

Based on the literature, individuals who practice outdoor sport-related activities receive high ultraviolet radiation exposure, have a high risk for skin cancer, have a high prevalence for pigmented lesions, and may benefit from electronic sun protection educational interventions.

**Conclusions:**

Individuals who practice outdoor sports experience substantially higher ultraviolet radiation exposure, routinely exceed the recommended exposure limits, and are at a higher risk of developing skin cancer. Therefore, those who are frequently engaged in outdoor leisure activities should be coached about efficient sun protective practices and relevant mobile technologies that may facilitate adherence.

## Key Points

- Individuals performing outdoor sports experience increased risk of skin cancer, increased prevalence of pigmented lesions in sun-exposed areas, and experience greater overall sun exposure.
- Modern technology utilizing the ultraviolet index as tool for modifying sun-protective behavior confers a mildly positive benefit.
- This evidence-based assessment supports the assumption of outdoor sportsmen and women being in greater need of sun-protective behavior counseling by their healthcare provider.

## Background

Skin cancer is the most common cancer in the USA. It is five times more common than breast or prostate cancer [1]. Moreover, skin cancer incidence is increasing. According to 2012 estimations, the number of patients diagnosed annually with non-melanoma skin cancer (NMSC) approaches 3.3 million, representing a 50% increase from 2006 [2, 3]. The 2020 *Annual Report to the Nation on the Status of Cancer* reveals an annual incidence of melanoma of 28.5 per 100,000 persons for men and 17.6 per 100,000 for women, which translates to a respective 5-year average annual percent change of 2.2% and 1.9% [4]. The WHO’s International Agency for Research on Cancer reports an age-standardized incidence rate (ASR) of all skin cancers at 68.1 per 100,000 persons in the USA. Elsewhere, such as Australia and New Zealand, the ASR is even higher at 181.1 and 176.1 skin cancers, respectively [5].

Outdoor sports athletes have high rates of sunburn [6–13] and low rates of skin cancer literacy [7, 14], thereby increasing their risk for cutaneous malignancy. A person’s orientation to the sun [15], the amount of sun exposure [16], and population behaviors [17, 18] toward sun exposure and protection may be determinant factors that explain the increasing frequency of skin cancer, but genetic, demographical, geographical, and meteorological differences make it difficult to predict an individual’s risk [18]. However, clinical recognition of high-risk behaviors can help identify those who need and will benefit most from individualized counseling on sun protective behaviors [18, 19].

It has been established that the number and severity of sunburns correlate with increased rates of melanoma later in life, with up to 90% attributed to UV exposure [18, 20]. While both UV-A (320–400 nm) and UV-B (290–320 nm) impact cutaneous health, UV-B is assumed to be the main culprit for inducing carcinogenic sequelae [16]. Equally as important to the risk of cancer is the health benefit of increased vitamin D levels associated with intermittent UV-B exposure [21, 22]. The relationship between systemic vitamin D levels and all-cause and specific-cause mortality has been documented in many studies [23–27]. The recommended exposures required to achieve the desired 25(OH)D levels are minimal in the summer months, although varying latitudes and weather conditions influence the time needed for adequate UV radiation exposure [28–30].

Despite the wealth of knowledge available to the public on sun care etiquette and skin cancer prevention, individuals continue to fail to engage in protective behaviors while outdoors [13, 31–33], and the melanoma incidence continues to rise [19]. Even in locations where UV-intensity remains elevated year-round, sun-protection programs lack widespread institutional adoption [17, 34]. These concerns emphasize the need for a greater understanding of how outdoor behavior impacts cutaneous health. Herein, we will review the strategies for determining personal UV exposure and evaluate the frequency of skin cancer and pigmented lesions in those who perform outdoor sport activities. In addition, we conducted a systematic review of current literature to evaluate the risk of developing skin cancer guided by the following research questions:

1. How is personal UVR measured?
2. What is the modern utility of the ultraviolet index in modifying recreational sun protection behaviors?
3. What is the risk of developing skin cancer for outdoor sport participants?
4. What is the prevalence of skin cancer in sport participants?
5. Is the number of nevi and solar lentigines elevated in sport participants?

## Methods

Query criteria and search terms are listed in Table 1. Our approach for each question is detailed as follows:

**Table 1:** Search terms

Search terms	
Database	Medline
Free text words	“ultraviolet index”^a^ “electronic dosimeters”^b^ “electronic sun journal”^c^ “Ultraviolet Index” OR “UV Index” OR “UVI” AND “Behavior”; “Mobile”; “Email”; “App”^d^ ((“skin cancer”/sports) OR “skin cancer” prevalence AND “sports”)^e^ “skin cancer” risk AND sports^f^ “Nevi Count AND Sports”^g^
Field	All fields^a–f^
Limits	Language: English^a–f^ Species: Human^a–f^ Time: None^a–d, g^ 01/1990–12/2018^e^ 03/1986–03/2019^f^
MeSH	Used^e^

Superscripts a–f denote the free text word search terms and their corresponding modifiers utilized in our Medline query

For question 1, we leveraged the *International Commission on Non-Ionizing Radiation Protection Statement against UVR* [35] and Schmalweiser and Siani’s *Review on Nonoccupational Personal Solar UV Exposure Measurements* [36]. We also performed focused searches using the terms “ultraviolet index” [37]. Finally, we explored approaches used to measure erythemally weighted UV irradiances accumulated over time on research participants based on references from Moehrle’s work [38] and additional focused Medline searches utilizing the terms “electronic dosimeters” and “electronic sun journal” [39–42]. A broader literature and citation search of all UVI measuring techniques yielded further sources [30, 35, 43–50]. For all other questions, we used Boolean text query strategies “AND”, “OR” on Medline. For question 2, we aimed to expand upon Italia et al.’s 2011 systematic review [51] on UVI interventions using technology by searching “Ultraviolet Index” OR “UV Index” OR “UVI” AND “Behavior” in the past 10 years, which resulted in 112 articles. Three relevant articles were found [52, 53] including the systematic review performed by Heckman et al. [54]. Citation searches yielded another publication [55]. Since question 2 is oriented to target electronic means of UVI communication for mobile athletes, we added various restrictions in place of “Behavior” including “Mobile”, “Email”, and “App”, yielding one relevant citation [56]. Additional focused searches discovered 3 more articles [57–59]. For questions 3–5, we evaluated studies from January 1990 through December 2018 utilizing the following search criteria in PubMed: ((“skin cancer”/sports) OR “skin cancer” prevalence AND “sports” [MeSH]). We identified 104 English publications related to humans, and 6 relevant records [60–65] were included for further review. Additional focused search yielded 6 articles [30, 66–71]. We expanded our search for question 3 by evaluating studies from March 1986 through March 2019 utilizing the following criteria in PubMed: “skin cancer” risk AND sports. Using this query, we found 67 publications related to humans written in English, of which we included 2 additional relevant records [72, 73]. For question 5, we specifically leveraged the work done by Richtig et al. [64], Ambros-Rudolph et al. [65], and Mahe et al. [69]. Of note, question 5 was added post hoc given that high densities of nevi have been associated with an increased risk of developing melanoma [69, 74, 75]. No additional records were found utilizing the following search criteria in PubMed: “nevi count AND sports.” The manuscript quality rating used in this review was based on the type of study, study sample size, and the relative strengths of outcomes measured.

## Results

**Fig. 1:**
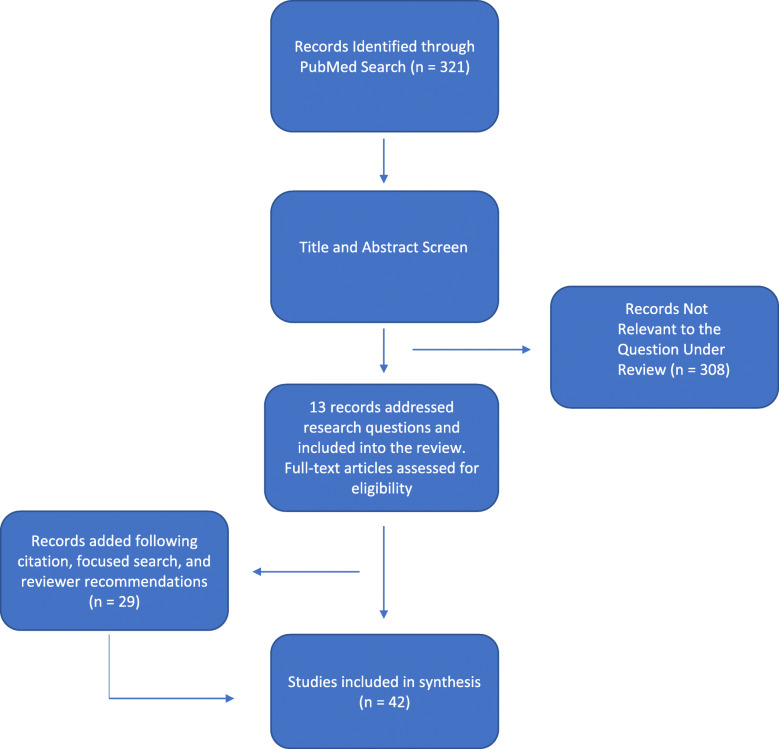
PRISMA flow chart of search strategy

Our search identified 321 records. After scanning titles and abstracts as described above, 13 studies were included for this systematic review (Fig. 1). A total of 29 records were added following citation search, focused search, and reviewer recommendations. A review of the included studies is listed below:

Question 1: How is personal UVR measured?

The ambient erythemal dose, or ambient exposure, is defined as the incident erythemally weighted irradiance on a horizontal surface (W/m^2^) over a specified period of time (J/m^2^). Accurate measurements of ambient doses can be performed by calibrated broad band radiometers or spectroradiometers [76]. For clinical studies, minimal erythema dose (MED) and standard erythema dose (SED) are the most common radiometric parameters. MED is the lowest UVR exposure sufficient to produce erythema within 8–24 h [35] and varies depending on the tanning and susceptibility to sunburn of each individual [43] (Supplementary Table 1). One SED has a set equivalent to an erythemal effective radiant exposure of 100 J/m^2^ using the C.I.E. action erythemal spectrum normalized to 298 nm [44]. In contrast to the MED, the SED measure is independent of skin type and is a more objective unit for the measurement of personal UV exposure (PE) via dosimetry [44, 45]. To put this in perspective, the International Commission on Non-Ionizing Radiation Protection (ICNIRP) recommends a daily occupational exposure limit (EL), defined as a maximum PE of 30 J m^−2^ (0.3 SED) within an 8-h time-frame for sensitive, unprotected skin [35].

Measuring UV exposure in the sports setting is complex; however, different dosimeters have been utilized to measure PE on a variety of platforms [45, 46]. Polysulfone plastic films [77, 78] and *Bacillus subtilis* spore films [79] are used as chemical and biological dosimeters, respectively. In addition, electronic UV dosimeters have been utilized [39–42], and electronic sun journals (ESJ) are available to track cumulative sun exposure [42]. PE quantified by SED can be utilized by researchers to track UV radiation over time, but individual measurements lack external validity due to different dosimeter orientations secondary to posture and varying environmental conditions [47, 48]. A more pragmatic calculation for comparing PE between sports is the exposure ratio to ambient (ERTA or ER). The ERTA is a ratio of PE relative to ambient UV radiation, which allows researchers to compare accurate dosimetry measurements across different settings while accounting for personal orientation, solar elevation, and other idiosyncratic confounders of precise UV exposure [36]. Since ratio changes can be relatively interpreted over time and across settings, it is a valuable method researchers can use to contrast UV exposure across sporting events in particular. The evolution of these UV radiation measurement technologies and inter-sport ERTA values is comprehensively described in the reviews of nonoccupational UV exposure by Schmalwieser and Siani [36] and Downs et al. [48].

While PE and ERTA are important quantitative measures for industries and researchers, their practical and primary preventative, non-research value to the public may be inhibited by the lack of dosimeter ubiquity, complex calibration requirements [46], and poor inter-reliability in comparison to meteorological grade instruments [45, 49]. The Global Solar Ultraviolet Index (UV Index, UVI, World Health Organization) [37] does not track cumulative PE; however, it is useful for predicting PE and erythemal skin damage risk. The UVI is calculated from the erythemally weighted UV irradiance by convolving the spectral irradiances (280–400 nm) with the spectral weighting function for erythema. It is individually interpreted by skin type and predisposition to sunburn [80] (Supplementary Table 2). Its unitless value can be quantified by the below equation:

$$ {I}_{UV}={k}_{er}\bullet {\int}_{250\mathrm{nm}}^{400\mathrm{nm}}{E}_{\lambda}\bullet {s}_{er}\left(\uplambda \right) d\lambda $$

where *E*_*λ*_ is the solar spectral irradiance expressed in *W*/(m^2^·nm^1^) at wavelength *λ* and d*λ* is the wavelength interval used in the summation. *s*_*er*_ is the erythema reference action spectrum, and *k*_*er*_ is a constant equal to 40 m^2^/W [37]. The integer output of the equation ranges from 1 to 11+, which provides individuals with a reference action spectrum for ultraviolet-induced erythema on human skin. Its primary role to the user is to serve as a numerical predictor of cutaneous damage from sun exposure. Moreover, the WHO has put forth scaled sun protection recommendations that complement the increasing risk of erythema or sunburn: 1–2, no protection required; 3–7, protection required; 8–11+, extra protection required [37]. By following these suggestions, individuals can reduce their risk of sunburn and thereby reducing their later risk of skin cancer.

The UVI is not an exact measure of ultraviolet exposure intensity, which varies with geographic location, solar altitude and angle, cloudiness, ozone thickness, aerosols, altitude, and surface albedo from adjacent surfaces such as water or snow [30, 50, 81]. It is measured or predicted by models using satellite-based instruments or from ground-level commercial radiation detectors, with the latter being more precise by being able to account for the aforementioned variables per location in real-time [46]. Geospecific UVIs are included in weather forecasts across many countries and integrated in smartphone applications [37, 82], making sun exposure prevention guidelines widely accessible to patients. During summer in the USA, the UV index can be either very high (8–10) or extreme (11+) at midday. The average UVI in July ranges from 6.5 at the continental US northernmost border to 11.5 in southern Texas [81]. For these conditions, the time needed to achieve erythema ranges from 12–15 min, based on conversions provided by the ICNIRP [35]. In the southern hemisphere, dangerously elevated UVIs are even more prevalent; extreme values of 20+ have been reported in the mountains of Hawaii, the Andes, and the Himalayas [81, 83]. Prior studies have noted increased skin cancer rates along the US latitudinal gradient [84]. Therefore, the UV index can be a powerful educational tool used to alert individuals about weather conditions permitting potentially damaging sun exposure while outdoors.

Question 2: What is the modern utility of the UVI in modifying recreational sun protection behaviors? (Supplementary Table 3)

Despite overwhelming scrutiny, the UVI has been adopted by many governments as the standard platform for public communication of UV exposure risk since its inception by the WHO 1994 [37]. Many of the research efforts detailing UVI knowledge have shown there is a minimal understanding of the UVI by the public [51, 54, 85–90] and that lower UVI value recommendations underpredict erythemal risk [91–93]. Medical professionals have similarly displayed limited knowledge or use of the UVI [94–96]. However, personal knowledge of the UVI may not be necessary to improve sun protection behaviors if mobile health technology can provide tailored recommendations on behalf of the individual. Thus, research examining the efficacy of technology-based interventions elicits the contemporary primary preventative value of the UVI in a different light.

Italia et al. [51] performed a thorough systematic review of the literature addressing this topic prior to 2011, approximately half a year before the iPhone 4S had been revealed [97]. They reviewed 25 studies that investigated the knowledge of, familiarity with, attitude towards, and behavioral impacts of the UVI in the public domain. In regard to familiarity, they reported that awareness of the UVI varied significantly across countries and that understanding of the index was minimal. They also found that the UVI had no impact on knowledge or attitude about UVR or skin cancer. Moreover, behavioral changes in response to UVI interventions were limited or nonexistent in the studies they reviewed. Heckman et al. [54] performed a recent systematic review (2019) of UVI-impact literature (*n* = 31) in which they compared research between countries. They also found sharp contrasts in UVI awareness between nations, with poor overall comprehension. Unlike Italia et al.’s review [51], Heckman et al. [54] found mixed results on UVI studies utilizing interventions, but stratification based on means of intervention was not addressed. One possible explanation for this change is the advancement and personalization of health technology over the past decade.

The proliferation of mobile and wearable technology and increased demand for electronic health information will continue to alter the UVI research landscape [98]. The number of connected devices worldwide has doubled since 2015 [99], and emerging generations have increasing levels of internet literacy [82]. From 2012 to 2015, the number of individuals who use portable electronic devices for accessing health information increased from 38 to 86% [100]. Moreover, there is public interest in receiving sun protection advice electronically [100, 101]. Since the studies covering this topic prior to 2011 have been extensively described, the following descriptions relate only to technology-based UVI interventions outside of the workplace published since 2011.

In 2015, Buller et al. [53] proposed the use of a mobile smartphone application, “Solar Cell,” to provide individuals with tailored data on UV exposure risk including the current and forecasted UVI. Participants in the intervention group increased use of shade when outdoors (41.0% vs 33.7%, *p* = 0.03) but reduced use of sunscreen (28.6% vs 34.5%, *p* = 0.48). People who used the app also reported a decreased average number of days in the sun (60.4% vs 49.3%, *p* = 0.04) and were more likely to use all sun protection behaviors combined (39.4% vs 33.8%, *p* = 0.04). No other significant associations between use of the app and sun protection habits were detected. Individuals in lower income brackets who used the app also displayed a greater confidence in sun protection strategies (*F* = 3.53, *p* = 0.01).

Their team performed an additional study [52] with a pretest-posttest design to investigate the effectiveness of SolarCell in altering sun protection habits. At the 7-week interim analysis, there was an increase in use of wide-brimmed hats among younger app users (23.8% vs 17.4%, *p* = 0.045), but the trend did not remain significant by the 12-week posttest analysis. No other associations with positive behavioral changes were found.

Buller et al. [58] performed a pair-matched pretest-posttest quasi-experimental study on the effect of the multi-component “Go Smart Sun” (GSS) educational intervention versus no intervention across 41 US resorts over 2 years. One component of the GSS educational campaign includes sharing the UV index to alert individuals to sun safety. The UVI was at least high (UVI > 5) in 55.5% of 3531 of the interviewed participants and 42.4% of the 4357 prospectively observed participants. In addition to printed materials, they shared sun protection education information via pre-arrival emails, social media messages, and videos that covered a wide range of sun safety techniques beyond UVI awareness. No differences were detected between arms. When stratified by venue type, waterside venues displayed improvements in sun protection behaviors per composite *z* score (*p* < 0.01).

Of note, Anderson et al. [59] analyzed the trends of sun protection behaviors in the baseline, pretest cohort. Although sun protection behaviors correlated most highly with increasing temperatures, they found that the UVI was significantly positively associated with sunscreen use and sunscreen reapplication in the retrospective sample (OR = 1.07, 1.19, *p* < 0.001). The relationship between UVI and shade use was positively significant in the observational sample (*β* = 0.01, *p* < 0.001), but UVI was negatively associated with clothing coverage (*β* = − 0.003, *p* = 0.004).

In 2016, a study on sun protection training of 26 adolescent organ transplant recipients via text messages was performed by Sachse et al. [55]. Initial in-person training of sun protection strategies that emphasized the utility of the UVI as a “sun protection traffic light” preceded 4 weeks of daily text reminders of the UVI traffic light forecast and behavioral recommendations. The pretest-posttest survey revealed an increased comprehension of the UVI (16% vs 74%), ABCDE mnemonic understanding (0% vs 37%), and recognition of sunburns being delayed from UV exposure onset (26% vs 47%). Sun avoidance behaviors related to redness (16% vs 5%) or warmth of skin (31% vs 31%) did not improve. At 8 weeks, 95% of patients read the messages daily and described the intervention as “very helpful.” Fifty-eight percent of participants reported changing their sun protection behaviors when the UVI was high, 53% increased sunscreen use, and 21% described protective clothing as more important relative to baseline.

Hacker et al. [57] performed a prospective study in 2018 comparing the effectiveness of a personal UVR dosimeter and a “SunSmart” mobile application in altering sun protection habits over 3 months. The app displayed the daily UVI, information on interpreting the UVI, the weather forecast, and a vitamin D tracker tool. Outcomes were measured on the validated Sun Protection Habits (SPH) scale. The SPH index increase was marginally higher in the app group than the UVR monitor group at the 3-month follow-up at + .14 and + .13, respectively, but differences between the two and the control group were nonsignificant. While the dosimeter arm was the only one to have a significant association between use and UV exposure (1-week reduced unprotected UVR exposure OR = 2.706, *p* = 0.04, 3-month 3.130, *p* = 0.02), the participants reported they found the app to be more encouraging and engaging (63% vs. 47%) and were more likely to download the app than to buy the dosimeter (40% vs. 19%).

A qualitative study comparing experiences of new (*n* = 45) and existing users (*n* = 15) of the SunSmart app was performed by Nicholson et al. [56] in 2019. They found that across groups there was a lack of comprehension of the UVI and that new users described the app’s recommendations as too prudent in comparison to their personal interpretations of daily risk. Importantly, they found that some existing users recognized their inability to gauge the daily UVI, and therefore relied on the SunSmart app to guide daily sun protection behaviors even though they also lacked comprehension of the UVI scale. The benefit was found mostly in individuals who adopted use of the app as part of their daily hygiene regimen.

Although the UVI was not a major component of their interventions, the Healthy Texts [102, 103], UV4.me [104], and Ho et al. [105] studies employed a similar method of technology-based educational interventions to improve sun protection behaviors. All displayed significant improvements in positive sun protection behaviors in the experimental populations (Supplementary Table 3). This underscores that behavior modification cannot be expected by sharing the UVI alone, but rather as an adjunct to other tailored information.

In summary, modern technology has enabled researchers to elicit mild to, at most, moderate sun-protective behavioral changes through electronic multimedia platform interventions. The UVI often serves as a referential crux for which personalized recommendations can be made rather than the impetus for change itself. Increased adherence to these sun protection strategies, as guided by the UVI scale, can decrease the risk of UV-induced erythemal damage to human skin and ultimately reduce the insidious risk of melanoma and other skin cancers attributed to excessive UV exposure. In future research, the utility of real-time UVR detection and UVI feedback in mobile and wearable devices has promising potential to guide patient heliotherapy and vitamin D exposure [82], predict and track UVR exposure at major sporting events [106], and enhance the sun-safety of individuals through extensive user-personalization [107].

Question 3: What is the risk of developing skin cancer in outdoor sports participants? (Table 2)

**Table 2:** Risk for outdoor sporting participants to develop skin cancer

Study	Type of study	Quality rating	Region	*N*	Outcomes	Hours of exposure
Rosso et al. “Helios”. II [66]	Case control	3	Southern Europe	1549 BCC, 228 SCC, 1795 controls	Significant association between BCC and water sports (swimming, surfing, boating, and sailing). Non-significant association between BCC and mountain sports (skiing, climbing, hiking) and air sports (flying, hang-gliding and parachuting). Non-significant association between SCC and water sports stronger with > 2112 h of exposure. Holidays at the beach OR 1.5 > 2464 cumulated hours Water sports: OR 1.5 > 771–2112 h	Risk of SCC = significantly increased at > 70,000 h of lifetime sun exposure Risk of BCC = 2-fold increase risk at 8000–10,000 cumulated hours in a lifetime
Rosso et al. [72]	Case control	3	Sion Switzerland	*n* = 146, controls = 144	Outdoor sports conveyed an increased risk for basal cell carcinoma: average OR 2.2 *p* = 0.05	288– > 3420 h of cumulated exposure
Schnohr et al. [67]	Cohort	4	Denmark	28,259 persons	Rate ratio 1.72 (95% CI 1.23–2.40; *p* = 0.001) for vigorous physical activity compared with low activity and non-melanoma skin cancer in men but not in women.	
Holman et al. [68]	Case control	3	Australia	507 melanoma patients, 507 age-, gender-, and location-matched controls	Boating increased risk for melanoma (OR = 2.43 *p* = 0.04) Fishing increased risk for melanoma OR = 2.72, *p* = 0.07 Whenever these sports were practiced one or more times a week	

Quality rating is based on the robustness of the type of study performed, sample size, and strength of the measured outcomes

*BCC* basal cell carcinoma, *SCC* squamous cell carcinoma, *OR* odds ratio, *CI* confidence interval

The multicenter south European study Helios II indicated that athletes participating in intense UVR exposure water sports such as swimming, surfing, boating, and sailing are at increased risk for development of BCC (odds ratio 1.6 for more than 2600 accumulated hours of exposure in a lifetime). Sports practiced in the mountains such as skiing, climbing, and hiking or in the air such as flying, hang-gliding, and parachuting had weaker or non-significant BCC association [66]. Zanetti et al.’s subanalyses of the Helios II data corroborated these trends by comparing the number of lifetime weighted hours against development of skin cancers. Although the results were not significant at *p* = 0.05, adjusted odds ratios for CMM, SCC, and BCC in outdoor sports and beach sports were still elevated at 1.5, 1.3, 0.9, and 1.2, 1.2, and 1.0 respectively [70]. Rosso et al. also found that outdoor sports participants had a twofold increase in risk of BCC with a borderline independent significance (*p* = 0.05) when evaluating a case control population from Switzerland [72].

Analyses performed by a Danish group revealed there was a significantly increased risk of non-melanoma skin cancer for men who participated in vigorous outdoor physical activities compared with those performing low-level physical activities (*p* = 0.001). No association was found in women [67]. With respect to melanoma, Moore et al. utilized data from twelve prospective studies and found that leisure time physical activity was associated with a higher risk of malignant melanoma. This association was found to be stronger in areas with high UV exposure [71]. Holman et al. found that participation in water sports such as boating had an increased risk of developing melanoma with an odds ratio (OR) of 2.43 (*p* = 0.04). Similarly, fishing had an increased risk with an OR of 2.72 (*p* = 0.07). These results applied whenever sports were practiced once or more per week [68].

Question 4: What is the prevalence of skin cancer in sport participants? (Table 3)

**Table 3:** Prevalence of pigmented lesions and skin cancer in participants in golf, cricket and surfing

Study	Quality rating	Sport	Region	*N*	Measures	Outcomes
del Boz et al. [73]	4	Golf	Spain	195	Physical examinations	Actinic keratosis was found in 40%; clinical suspicion of BCC in 7.7% Atypical nevi in 7.7%, SCC in 2.1%, melanoma in 1.5%
Noble-Jerks et al. [62]	4	Cricket	Australia	164	Questionnaires about lifetime diagnosis of skin cancer	38.4% (63) had at least one skin cancer
Dozier et al. [63]	3	Surf	Texas, USA	49 surfers, 60 controls	Physical examinations	AK 20 surfers; 8 controls (not significant) Atypical nevi 18 surfers; 6 controls (not significant) BCC 8 surfers; 1 control *p* < 0.047
Climstein et al. [60]	4	Surf	Australia	1348	Questionnaires about lifetime diagnosis of skin cancer	184 (13.6%) participants reported skin cancer. Higher relative risk (*p* < 0.001) in competitive vs recreational surfers (odds ratio 1.74 (CI 1.28–2.31)). BCC was the most frequent skin cancer reported (6.8%), followed by melanoma (1.4%) and SCC (0.6%)
Zink et al. [61]	4	Ski guides	Switzerland	62	Physical examinations	22 (35.4%) AK, 4 BCC (6.4%), 1 SCC (1.6%)

Quality rating is based on the robustness of the type of study performed, sample size, and strength of the measured outcomes

*BCC* basal cell carcinoma, *SCC* squamous cell carcinoma, *AK* actinic keratosis

In a study by del Boz, physical examinations were conducted in 195 golfers: actinic keratosis was found in 40%, atypical nevi in 7.7%, clinical suspicion of melanoma in 1.5%, suspicion of SCC in 2.1%, and suspicion of BCC in 7.7% [73]. When former Australian male cricket players (*N* = 164) responded to questionnaires about lifetime diagnosis of skin cancer, 38.4% (*n* = 63) of respondents had been diagnosed with at least one skin cancer. Twenty-three responders with histories of skin cancer indicated that they either occasionally, very rarely, or never used at least 2 of the 3 recommended skin protection strategies (wearing a wide-brimmed hat, long-sleeved shirt, and the use of sunscreen) [62]. Zink et al. performed a cross-sectional analysis of skin cancer in 62 mountain and ski guides in Switzerland via physical examination. 43.5% (*n* = 27) was diagnosed with malignant lesions including AK (*n* = 22, 35.4%), BCC (*n* = 4, 6.4%), and SCC (*n* = 1, 1.6%) [61].

Dozier et al. performed a skin cancer screen of 49 surfers during a competition in Texas via physical examination. Investigators found 8 BCCs on surfers whereas only 1 BCC was found in the control group (*p* < 0.047) [63]. Australian investigators performed surveys on 1348 recreational and competitive surfers for lifetime prevalence of skin cancer, of which 184 (13.6%) participants reported skin cancer. The relative risk of developing skin cancer was significantly higher (*p* < 0.001) in competitive vs recreational surfers (odds ratio 1.74 (CI 1.28–2.31). BCC was the most frequent skin cancer reported (6.8%), followed by melanoma (1.4%) and SCC (0.6%) [60].

Question 5. Is the number of nevi and solar lentigines elevated in sport-participants? (Supplementary Table 4)

Richtig et al. [64] and Ambros-Rudolph et al. [65] performed assessments on 150 and 210 marathon runners, respectively. Both studies revealed a significantly elevated number of nevi, atypical nevi, and solar lentigines in marathon runners. Richtig et al. found 19.6 ± 18.2 lentigines on the shoulder when compared to 0 lentigines on the buttocks in the same group. Runners reporting more than 10 lifetime sunburns had more lentigines on their shoulder (*p* = 0.032). The mean number of counted nevi on the left shoulder was 1.3 ± 2.1 compared to 0.5 ± 1.0 on the left buttocks (*p* = 0.000). Ambros-Rudolph found 99 runners with more than 1 atypical nevi compared with 66 in the control group (*p* = 0.001). An increased number of solar lentigines was found in 64 marathon runners when compared to the 42 participants in the control group (*p* = 0.01). Another study [69] assessed melanocytic nevi count on children who practiced outdoor sports compared to those who did not. Investigators found a mean of 17.2 nevi on those who practiced outdoor sports and a mean of 15 nevi on those who did not (*p* < 0.001). When gender differences were assessed, boys were found to have significantly increased nevi count on the back area whereas girls did not show an increased number of nevi on the back (Supplementary Table 4).

## Discussion

In this review, we identified a number of methods used to measure solar UVR and discussed the applicability of these measurement tools in the personal and research settings. We also found that, while awareness of the UVI varies significantly and comprehension is low even after intervention, access to an electronic tool that provides preventative sun protection behavior recommendations may be successful in altering habits, possibly by means of instruction rather than teaching. Lastly, we addressed that individuals who practice sport-related activities have a higher risk for skin cancer and higher prevalence for pigmented lesions in sun-exposed areas.

Strengths of this study include a longitudinal and focused review of UVR risk assessment tools and outcomes in a defined population, a comprehensive literature search, and the identification of contemporary interventions to improve upon current sun-safety recommendations. Limitations are narrow inclusion criteria and consequent requirement for extensive citation search, the lack of a validated manuscript appraisal scale, and the inherent biases of data included from observational behavioral studies incorporated in the review.

This evidence-based assessment supports the assumption of outdoor sportsmen and women being in greater need of sun-protective behavior counseling by their healthcare providers. Therefore, we highlight here the national guidelines outlined by the Surgeon General in an effort to prevent skin cancer [6]. Emphasis is placed on the individual to adopt protective behavioral strategies such as wearing tightly woven long clothing, hats, and sunglasses; using at least 15 SPF sunscreen before outdoor activity; seeking shade; and avoiding being outside during hours of peak UV intensity. In particular, adolescents and young adults are considered vulnerable but impressionable. Clinicians are recommended to perform tailored, brief interventions in this demographic. Lastly, the guidelines call for legislative involvement at the local, state, and federal level to expand educational programs as well as enable access to proper protective clothing and shade in the workplace and on campuses nationwide [6].

Current literature corroborates these prudent recommendations, especially for athletes [7, 11, 13]. Recent cross-sectional studies [108–110] provide evidence to discourage the use of sunscreen as the only sun protection strategy [111]. Athlete-specific educational interventions have been shown to be effective, such as the SUNSPORT with NCAA student-athletes [112]. Sharing these strategies seems to be most effective when incorporated as part of a multi-component intervention rather than mass media interventions alone [113]. Since the 1980s, educational initiatives such as “slip, slop, slap, seek, slide” have been implemented in other parts of the world [51] to educate about the use of protective clothing, sunscreen, and broad brimmed hats and the importance of seeking shade and wearing sunglasses. However, in order to maintain generational relevance, it should be stressed that the modern ubiquity of mobile technology can complement this multidecade-old adage. The fact that the SunSmart App has been downloaded 300,000 times is a testament to the success of the modernization of Australia’s famously successful public health effort [56]. Outside of the research setting, the UVI remains a core component of personalizing sun-safety communication in mobile apps designed for commercial use in the USA [114]. Patient counseling on the availability and utility of these resources may help individuals adopt sun-safe hygienic routines before or during outdoor sports, regardless of whether their understanding of the UVI is improved or not.

Lastly, Bloom et al. [115] demonstrated increased interest in skin cancer in the population during summer months; as such, educational campaigns may be most effective when the population is more receptive and actively seeking information during the summertime. In summary, a timely educational program that optimizes the core principles of historically successful programs with avant-garde technology may elicit the greatest results in coaching sun protective habits.

## Conclusion

Individuals involved in outdoor daytime activities experience substantially high UVR exposure but continue to misunderstand the public utility of the UVI. In addition, they are at high risk of developing skin cancer. Therefore, clinicians should provide preventative counseling and educational support on sun-protection strategies in this high-risk population. We recommend the use of the following sun protection approaches: seeking shade, wearing protective clothing, and using sunscreen while discouraging the use of sunscreen as the only sun-protection strategy. Smartphones and wearable technology with apps that provide UVR avoidance instructions may help athletes adhere to proper protective behaviors before and during outdoors activities. It remains necessary to investigate UVR exposure with newer technologies to more accurately evaluate the contribution of UVR exposure to skin cancer.

## Supplementary information

**Additional file 1: Table S1.** “Number of SED required to induce erythema according to skin phototype” Adapted from International Commission on Illumination^22^.

**Additional file 2: Table S2.** “Comparison of time needed to exceed ICNIRP threshold and to achieve erythema with respective UV index for the different non-adapted skin phototypes”.

**Additional file 3: Table S3.** Comparison of studies using modern technology to improve sun protection behaviors.

**Additional file 4: Table S4.** “Prevalence of lentigines and nevi on marathon runners and children who practice outdoor sports”.

## Data Availability

Not applicable.

## Abbreviations

ICNIRP: International Commission on Non-Ionizing Radiation
C.I.E.: International Commission on Illumination
UV: Ultraviolet
UVR: Ultraviolet radiation
UVI: Ultraviolet index
WHO: World Health Organization
MED: Minimal erythemal dose
SED: Standard erythemal dose
PE: Personal exposure
ERTA: Exposure-to-ambient ratio
OR: Odds ratio
CMM: Cutaneous malignant melanoma
SCC: Squamous cell carcinoma
BCC: Basal cell carcinoma
