# Supplementary material for: Solar Ultraviolet Exposure in Individuals Who Perform Outdoor Sport Activities
Source: Sports Med Open. 2020 Sep 3;6:42. doi: 10.1186/s40798-020-00272-9 (PMC7471243; doi:10.1186/s40798-020-00272-9)
Supplement: Supplementary file 1 — Additional file 1: Table S1. “Number of SED required to induce erythema according to skin phototype” Adapted from International Commission on Illumination22. [file 40798_2020_272_MOESM1_ESM.docx]

Supplemental Table 1. “Number of SED required to induce erythema according to skin phototype” Adapted from International Commission on Illumination^22^

| Skin phototype | Skin without adaptation | Skin with adaptation |
| --- | --- | --- |
| I – II | 2 SED | 5 SED |
| III-IV | 5 SED | 12 SED |
| V | 10 SED | 60 SED |
| VI | 15 SED | 80 SED |

SED: Standard erythemal dose
