# Supplementary material for: Solar Ultraviolet Exposure in Individuals Who Perform Outdoor Sport Activities
Source: Sports Med Open. 2020 Sep 3;6:42. doi: 10.1186/s40798-020-00272-9 (PMC7471243; doi:10.1186/s40798-020-00272-9)
Supplement: Supplementary file 2 — Additional file 2: Table S2. “Comparison of time needed to exceed ICNIRP threshold and to achieve erythema with respective UV index for the different non-adapted skin phototypes”. [file 40798_2020_272_MOESM2_ESM.docx]

Supplemental Table 2. “Comparison of time needed to exceed ICNIRP threshold and to achieve erythema with respective UV index for the different non-adapted skin phototypes”

| UV index | | Time to exceed ICNIRP exposure limit  (min) | Time to achieve erythema (min) | | | |
| --- | --- | --- | --- | --- | --- | --- |
|  |  |  | Skin phototype I, II (2 SED) | Skin phototype III, IV (5 SED) | Skin phototype V (10 SED) | Skin phototype VI (15 SED) |
| Low | 1 | 79.3 | 133 | 333 | 667 | 1000 |
|  | 2 | 39.7 | 67 | 167 | 333 | 500 |
| Moderate | 3 | 26.4 | 44 | 111 | 222 | 333 |
|  | 4 | 19.8 | 33 | 83 | 167 | 250 |
|  | 5 | 15.9 | 27 | 67 | 133 | 200 |
| High | 6 | 13.2 | 22 | 56 | 111 | 167 |
|  | 7 | 11.3 | 19 | 48 | 95 | 143 |
| Very high | 8 | 9.9 | 17 | 42 | 83 | 125 |
|  | 9 | 8.8 | 15 | 37 | 74 | 111 |
|  | 10 | 7.9 | 13 | 33 | 67 | 100 |
| Extreme | 11 | 7.2 | 12 | 30 | 61 | 91 |
|  | 12 | 6.6 | 11 | 28 | 56 | 83 |
|  | 13 | 6.1 | 10 | 26 | 51 | 77 |
|  | 14 | 5.7 | 10 | 24 | 48 | 71 |

ICNIRP: International Commission on Non-Ionizing Radiation Protection; UV Index: ultraviolet index. SED: standard erythemal dose; min: minutes.
