# Supplementary material for: Solar Ultraviolet Exposure in Individuals Who Perform Outdoor Sport Activities
Source: Sports Med Open. 2020 Sep 3;6:42. doi: 10.1186/s40798-020-00272-9 (PMC7471243; doi:10.1186/s40798-020-00272-9)
Supplement: Supplementary file 3 — Additional file 3: Table S3. Comparison of studies using modern technology to improve sun protection behaviors. [file 40798_2020_272_MOESM3_ESM.docx]

Supplemental Table 3. Comparison of studies using modern technology to improve sun protection behaviors.

| Authors | Study Type | Population | Quality rating | Country | Technology & Methods | Intervention | Findings |
| --- | --- | --- | --- | --- | --- | --- | --- |
| Italia et al.^51^  (2011) | Systematic Review | 25 studies | 5 | n/a | n/a | N/A | -Low to intermediate levels of UVI awareness  -UVI has no effect on knowledge, attitude and limited to no impact on sun exposure of sun protection behaviors |
| Heckman et al.^54^ (2019) | Systematic Review | 31 studies | 5 | n/a | n/a | N/A | -High levels of awareness in USA, Canada, NZ, Australia. Low awareness in Europe and others.  -Mixed results on behavioral interventions with UVI |
| Buller et al.^53^ (2015) | RCT | 604  adults | 4 | USA | Mobile App vs Control  Posttest-pretest with follow-up at 10 weeks | SolarCell App: hourly UVI, sun protection advice, risk alerts, VitD tracking) | -Increased shade (41% vs 33.7%, p = 0.03)  -Decreased sunscreen (28.6% vs 34.5, p = 0048)  -Decreased sun exposure (60.4% vs 49.3%, p = 0.04)  -Increased use of all protective behaviors combined (39.4% vs 33.8%, p = 0.04)  -No significant association with sunburns |
| Buller et al.^52^  (2015) | RCT | 202  adults | 4 | USA | Mobile App vs control  Posttest-pretest with follow-up at 7 and 12 weeks | SolarCell App: hourly UVI, sun protection advice, risk alerts, VitD tracking) | -Increased use of wide-brim hats at 7 wks (23.8% vs 17.4%, p = 0/045)  -Women increased use of all protective behaviors combined more than men (46.4% vs 43.3%, p = 0.04). -Men and older cohort used less sunscreen (32.7 vs 35.5%, p = 0.02) and hats (15.6% vs 17.9%, p = 0.03) |
| Buller et al.^58^  (2016) | Pair-matched, group-randomized, quasi-experimental | Pretest = 4347  Posttest = 3986 | 3 | USA/Canada | Print, audiovisual, online messaging  Pretest-posttest cross-sectional observations | GSS: Sun-safety, UVI information on posters, printed materials, emails, newsletter articles, and social media | -No significant differences in sun protection habit per experimental condition or intervention.  -Stratification by (+)/(-) waterside proximity revealed increased protection habits per z-score; p < 0.01  -Intervention (+) : pre = -22.74, post = -15.77; Control (+) : pre = -27.24, post = -23.24  -Intervention (-) : pre = 20.43, post = 20.53  Control (-) : pre = 22.94, post = 18.03 |
| Anderson et al.^59^  (2016) | Pair-matched, group-randomized Cross-sectional observation | Interviewed = 3531  Observed  = 4347 | 3 | USA/Canada | No intervention.  Cross sectional analysis of 2016 Buller et al. baseline population. | N/A | -Sun protection behaviors correlated most with increasing temperature  -UVI level was weakly associated with sun protection behaviors in the interview cohort  (OR = 1.07, 1.19, p < 0.001)  - UVI and shade use were positively significant in the observational sample (β = 0.01, p < 0.001) but UVI was negatively associated with clothing coverage (β = -0.003, p = 0.004) |
| Sasche et al.^55^  (2016) | Pretest-posttest intervention with no control | 26 organ transplant recipients, age 13-22 | 3 | Germany | In-person training, 4 weeks of daily text messages  Pre/post questionnaires at wk 4 and interview wk 8 | HIPPOlino: 5-hour face-to-face training on skin cancer prevention with UVI “traffic light” counselling, daily text messages with daily UVI traffic light, weather forecast, and sun-protection suggestions | -Increased UVI comprehension (16% vs 74%)  -Increased ABCDE mnemonic understanding (0% vs 37%  -Increased recognition of sunburns being delayed from UV exposure onset (26% - 47%).  -58% reported a change in protective behavior when UVI was high  -53% reported increased sunscreen reapplication  -21% reported increased importance of protective clothing  -No changes in awareness of sunburn related to skin warmth or redness |
| Hacker et al.^57^  (2018) | RCT | 124 young adults | 4 | Australia | UVR dosimeter, Mobile App  4-wk intervention of UVR monitor, SunSmart App, control groups for with follow-up at 1 wk and 3 mos | No device, UVR monitor with feedback, and SunSmart app (daily UVI, forecast, VitD tracker, protection suggestions) groups competed daily sun diaries during the 4 weeks intervention | -No significant difference between in change in Sun Protection Habits Index across the three groups (App = +0.14, UVR monitor +0.13)  -UVR monitor group reduced unprotected weekend exposure (1 wk OR = 2.706, p = 0.04; 3-mo OR 3.130, p = 0.02)  -Users found the app more encouraging and engaging than the dosimeter (63% vs 47%) and were more likely to download it than buy the dosimeter (40% vs 19%) |
| Nicholson et al.^56^  (2019) | Qualitative Survey | 45 new, 15 existing SunSmart users | 2 | Australia | Mobile App  Cross-sectional survey to elicit opinions of SunSmart App | Identification of semantic themes on use of the SunSmart App: Daily UVI and weather forecast, VitD tracker, Sun protection recommendations | -New users rarely apply their learning from the app to change sun protective behaviors and did not find the predictions to align with their UV risk expectations  -Existing users accepted their inability to gauge UV risk appropriately and incorporated the app into their daily routine to guide protective behaviors |
| Youl et al.^103^  (2015) | RCT | 546 young adults | 5 | Australia | Text Messaging  3 groups (self-examination, sun protection, attention control) received 21 texts over 1 yr with telephone surveys at 3 and 12 mos | Healthy Texts: Each group received topic-specific reminders and health guidance to encourage positive behaviors. UVI was not a major component of the program. | -At 1 yr, sun protection group improved from baseline (+0.12, p = 0.03) relative to control  -At 1 yr, skin self-examination improved from baseline (+.12, p = 0.035) relative to control  -No significant effect on tanning, sunburn behaviors or whole-body skin exams. |
| Heckman et al.^104^  (2016) | RCT | 1,234  adults | 4 | USA | Website  3 groups (assessment only, skin, skin cancer org website, or UV4.me program) assessed at 3 and 12 weeks post baseline | UV4.ME: Twelve, interactive modules on topics deemed important for sun protective behavior. The UVI was not explicitly labeled as a major component of the program. | -Significant decreases in UV exposure at 3 and 12 wks (cohens d = 0.41, 0.43; p < 0.001)  -Significant increases in skin protection at 3 and 12 wks (cohens d = 0.41, 0.53; p < 0.001) |
| Ho et al.^105^  (2016) | RCT | 300 caregiver-child pairs | 4 | USA | Text Messaging,  Intervention (daily texts, read-along book, swim shirt) vs control (basic well child visit counseling) with a 4 wk follow-up | Multicomponent intervention utilizing text messaging 4 times per week providing sun protection advice to the caregiver. The UVI was not a major component of this program. | -Interventions groups had higher sun protection behaviors on sunny and cloudy days (p < 0.01)  -Spectrophotometry revealed significant increases in melanin levels in the control group (p < 0.008) but not in the intervention group |

UVI, Ultraviolet Index; UVR, Ultraviolet radiation; OR, odds ratio; wk, week; mo(s), month(s); yr, year; USA, United States of America; NZ, New Zealand; VitD, Vitamin D; SED, standard erythemal dose; N/A, not applicable.

Quality rating is based on the robustness of the type of study performed, sample size, and strength of the measured outcomes.
