# Supplementary material for: Solar Ultraviolet Exposure in Individuals Who Perform Outdoor Sport Activities
Source: Sports Med Open. 2020 Sep 3;6:42. doi: 10.1186/s40798-020-00272-9 (PMC7471243; doi:10.1186/s40798-020-00272-9)
Supplement: Supplementary file 4 — Additional file 4: Table S4. “Prevalence of lentigines and nevi on marathon runners and children who practice outdoor sports”. [file 40798_2020_272_MOESM4_ESM.docx]

Supplementary table 4. “Prevalence of lentigines and nevi on marathon runners and children who practice outdoor sports”.

| Study | Quality rating | Sport | N | Measures | Outcomes |
| --- | --- | --- | --- | --- | --- |
| Richtig et al.^64^ | 4 | Marathon runners | 150 | Skin exam | Number lentigines on shoulder was 19.6 +/- 18.2 (SD), whereas no lentigines were found on the left buttock (p = 0.000). Number of nevi on shoulder 1.3 ± 2.1 compared to 0.5 ± 1.0 on left buttock (P=0.000) |
| Ambros-Rudolph et al.^65^ | 4 | Marathon runners | 210 | Skin exam | >50 melanocytic nevi 29 marathon group (MG) vs 47 in control group (CG) >1 atypical nevus 99 MG vs 66 in CG Numerous solar lentigines 64 MG vs 42 in CG |
| Mahe et al.^69^ | 3 | children who practice outdoor sports | 344  316 controls | Skin exam number of nevi | 22.1 nevi on those who practiced outdoor sports vs a mean of 19.9 on those who did not (P<0.012). When gender differences were assessed, boys were found to have significantly increased nevi count on the back |

SD, standard deviation; MG, marathon group; CG, control group

Quality rating is based on the robustness of the type of study performed, sample size, and strength of the measured outcomes.
